# Tigecycline Therapy for Nosocomial Pneumonia due to Carbapenem-Resistant Gram-Negative Bacteria in Critically Ill Patients Who Received Inappropriate Initial Antibiotic Treatment: A Retrospective Case Study

**DOI:** 10.1155/2016/8395268

**Published:** 2016-12-04

**Authors:** Xiaomai Wu, Yefei Zhu, Qiuying Chen, Liuyang Gong, Jian Lin, Dongqing Lv, Jiaxi Feng

**Affiliations:** ^1^Department of Respiratory Medicine, Taizhou Hospital of Zhejiang Province, Linhai 317000, China; ^2^Operation Department, Taizhou Hospital of Zhejiang Province, Linhai 317000, China

## Abstract

*Background*. Nosocomial pneumonia due to carbapenem-resistant Gram-negative bacteria (CRGNB) is a growing concern because treatment options are limited and the mortality rate is high. The effect of tigecycline (TGC) on nosocomial pneumonia due to CRGNB in patients who have received inappropriate initial empiric antibiotic treatment (IIAT) is unclear. Therefore, this study aimed to examine the effect of TGC on nosocomial pneumonia due to CRGNB in critically ill patients who had received IIAT.* Methods*. A retrospective study was conducted in an adult respiratory intensive care unit. Data were obtained and analyzed for all patients who were treated with TGC ≥ 3 days for microbiologically confirmed nosocomial pneumonia due to CRGNB and had experienced initial antibiotic failure. Clinical and microbiological outcomes were investigated.* Results*. Thirty-one patients with hospital-acquired pneumonia or ventilator-associated pneumonia were included in the study. The majority of the responsible organisms were carbapenem-resistant* Acinetobacter baumannii* (67.7%), followed by* Klebsiella pneumoniae* (16.1%) and* Escherichia coli* (9.7%). Twenty patients were treated with high-dose TGC therapy (100 mg every 12 h after a 200 mg loading dose), and the others received a standard-dose therapy (50 mg every 12 h after a 100 mg loading dose). The duration of TGC therapy was 14.3 ± 2.8 days. The global clinical cure rate and the microbiological eradication rate were 48.4% and 61.3%, respectively. The overall ICU mortality rate was 45.2%. A higher score on the Acute Physiology and Chronic Health Evaluation II and a longer duration of IIAT were associated with clinical failure. High-dose TGC therapy had a higher clinical success rate [65.0% (13/20) versus 18.2% (2/11), *P* = 0.023] and a lower ICU mortality rate [30.0% (6/20) versus 72.7% (8/11), *P* = 0.031] than the standard-dose therapy.* Conclusions*. TGC, especially a high-dose regimen, might be a justifiable option for critically ill patients with nosocomial pneumonia due to CRGNB who have received IIAT when the options for these patients are limited.

## 1. Introduction

Tigecycline (TGC) is a derivative of tetracycline that is also known as glycycline and that has a broad spectrum of activity [1]. In vitro, TGC exhibits excellent antibacterial activity against most aerobic and anaerobic bacteria, including multidrug-resistant (MDR) and extensively drug-resistant organisms [2–5]. However, a recent systematic review and meta-analysis suggested that TGC is also associated with a higher risk of death than other antibiotics [6, 7]. The Food and Drug Administration has therefore warned clinicians about the risks of TGC.

Despite these limitations, because of the scarcity of other effective antimicrobials, TGC has been a useful alternative in patients with serious infections caused by MDR organisms, such as carbapenem-resistant Gram-negative bacteria (CRGNB) [8–12].

Nosocomial pneumonia caused by CRGNB continues to be a growing concern, particularly in the intensive care unit (ICU), because it has limited treatment options and is associated with a high mortality rate [13–15]. There is a high risk of failure when using initial empiric antibiotic treatments for infections with CRGNB because the pathogens are usually not covered [14, 16, 17]. Although TGC exhibits considerable in vitro efficacy against CRGNB, the clinical effects of TGC in severe infections caused by CRGNB are controversial [2–7].

The effect of TGC on nosocomial pneumonia due to CRGNB in critically ill patients who have received inappropriate initial empiric antibiotic treatment (IIAT) has not been previously reported. Therefore, this retrospective clinical case study was aimed at assessing the effect of TGC on these patients.

## 2. Materials and Methods

This study was conducted in an eight-bed adult respiratory ICU in a tertiary care hospital and was approved by the Institutional Medical Ethics Review Board of Taizhou Hospital of Zhejiang Province. The need for informed consent was waived because of its retrospective design.

All of the patients included in this study were admitted to the respiratory ICU between 1 January 2013 and 31 December 2015. The inclusion criterion was the administration of TGC for microbiologically documented nosocomial pneumonia caused by CRGNB after any failed initial empiric antibiotic treatment. TGC treatment needed to be provided for at least 3 days.

The data were collected from patient medical records and hospital databases. Clinical data included demographics, underlying diseases, clinical and laboratory findings, microbiological results, Acute Physiology and Chronic Health Evaluation II (APACHE II) scores, and Sequential Organ Failure Assessment (SOFA) scores at admission to the ICU. Data related to information regarding the type and duration of antibiotics used before TGC treatment, the dose and duration of TGC therapy, concomitant antibiotics, clinical and microbiological responses, and ICU mortality were also collected.

### 2.1. Definitions

In this study, nosocomial pneumonia included hospital-acquired pneumonia (HAP), healthcare-associated pneumonia, and ventilator-associated pneumonia (VAP), which were defined according to the guidelines of the American Thoracic Society and Infectious Diseases Society of America [18]. A diagnosis of pneumonia was made on the basis of abnormal radiographic results (including new or persistent focal infiltrates or a diffuse lung injury pattern) along with one or more of the following criteria: (1) fever or leukocytosis/leukopenia; (2) respiratory failure requiring mechanical ventilation; or (3) at least two of the following: cough, dyspnea, tachypnea, pleuritic chest pain, auscultatory findings of rales or evidence of consolidation, hypoxemia, and purulent sputum or a change in sputum characteristics.

IIAT was defined as antibiotic therapy administered prior to a determination of the infectious pathogens and a later determination that the pathogens were uncovered by the antibiotics. Failed initial empiric antibiotic treatment was defined as meeting one or more of the following criteria after ≥48 h of treatment: (1) the clinical manifestations of pneumonia were not improved; (2) respiratory failure or other organ dysfunction was present or worsened due to pneumonia, (3) laboratory tests indicated deterioration of leukocyte, neutrophil, C-reactive protein, and procalcitonin levels; or (4) a chest radiograph showed progression. Microbiological eradication was defined as negative culture results for the original pathogens during or after the course of TGC therapy.

Responses to treatment were defined as either clinically successful or unsuccessful. Successful treatment was defined as the complete resolution of the infection-related abnormalities by the end of TGC therapy. Improvement in signs on chest radiography was also required for VAP. The criteria for unsuccessful treatment included the persistence of the initial signs of infection that required a change in antibiotic therapy, the reappearance of the initial signs of infection, or infection-related death.

### 2.2. Microbiology Analysis

Quantitative or semiquantitative bacterial cultures were obtained from the bronchoalveolar lavage (BAL), bronchoscopic aspirate, endotracheal aspirate, or qualified sputum specimens. Strains and antimicrobial susceptibilities were identified using the VITEK® 2 system (bioMérieux, Marcy-l'Etoile, France). The Clinical and Laboratory Standards Institute criteria published in 2012 were used to interpret the results. CRGNB was defined as carbapenem-nonsusceptible (MIC ≥ 2 mg/L) and extended-spectrum cephalosporin-resistant Gram-negative bacteria. With regard to tigecycline, susceptibility was interpreted according to the breakpoints approved by the US Food and Drug Administration. The isolates were considered susceptible to TGC if the MIC was ≤2 mg/L, intermediate if the MIC = 4 mg/L, and resistant if the MIC ≥ 8 mg/L.

### 2.3. Statistical Analysis

The Shapiro–Wilk test was used to evaluate the distribution of variables. Continuous variables were assessed using Student's *t*-test or the Mann–Whitney *U*-test, as appropriate, and expressed as the mean ± standard deviation (SD) or median and interquartile range (IQR). Categorical variables were analyzed using Fisher's exact test and are presented as proportions. A *P* value < 0.05 was considered statistically significant. The SPSS v16.0 package (SPSS Inc., Chicago, IL, USA) was used for all statistical analyses.

## 3. Results

### 3.1. Demographic Characteristics of the Patients

**Figure 1:**
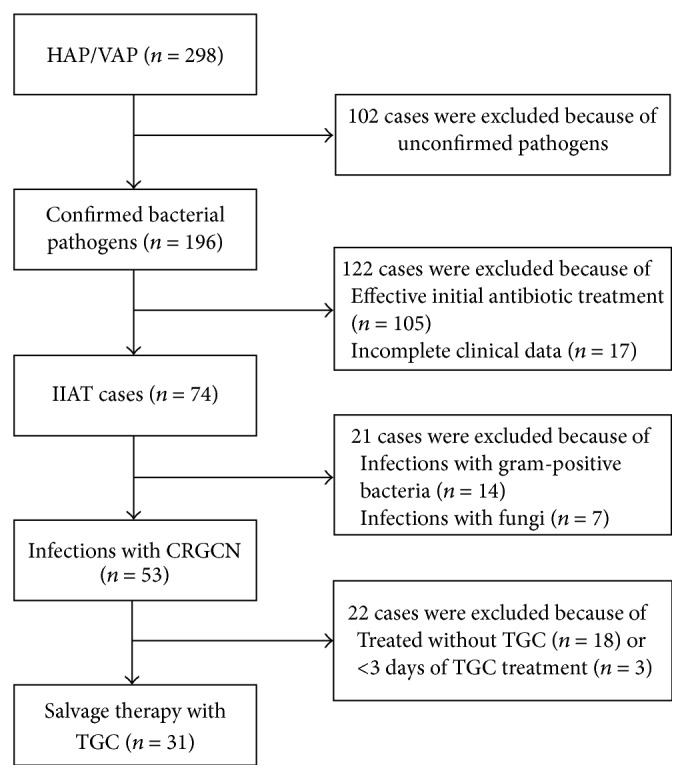
Study analysis populations. HAP: hospital-acquired pneumonia, VAP: ventilator-associated pneumonia, IIAT: inappropriate initial empiric antibiotic treatment, CRGNB: carbapenem-resistant Gram-negative bacteria, and TGC: tigecycline.

During the study period, 1085 patients were admitted to the respiratory ICU. Two hundred and ninety-eight patients were diagnosed with HAP, including 95 patients with VAP. The bacterial pathogens were confirmed in 65.8% (196/298) of these cases, and the initial empirical antibiotic treatment was found to be inappropriate in 37.8% (74/196) of the patients. Patients infected with CRGNB accounted for 71.6% (53/74) of the cases with IIAT. Among these, 31 cases that were treated with TGC as a salvage therapy were included in the study (Figure 1). The demographics and clinical characteristics of the study patients are shown in Table 1.

### 3.2. Microbiological Characteristics and Antibiotic Regimens

Among the 31 included patients, the most frequent causative organisms were* Acinetobacter baumannii* (*n* = 21, 67.7%) and* Klebsiella pneumoniae* (*n* = 5, 16.1%), followed by* Escherichia coli* (*n* = 3, 9.7%) and* Stenotrophomonas maltophilia* (*n* = 2, 6.5%). The antimicrobial resistances of the isolates are shown in Table 2.

Carbapenem (*n* = 15, 48.4%) was the most frequently used initial empiric antibiotic treatment in the cohort, followed by a *β*-lactam/*β*-lactamase inhibitor combination and fluoroquinolone (Table 1). The median duration of IIAT was 13 days (IQR: 7–20 days).

Twenty patients were treated with TGC at 100 mg every 12 h after a 200 mg loading dose, and the other 11 patients were treated with TGC in an initial loading dose of 100 mg followed by 50 mg every 12 h. Except for one patient who was treated with TGC monotherapy, the other 30 patients received combination therapy with cefoperazone-sulbactam (*n* = 18, 58.1%), piperacillin-tazobactam (*n* = 10, 32.3%), or carbapenem (*n* = 3, 9.7%). The mean duration of TGC therapy was 14.3 ± 2.8 days.

### 3.3. Clinical and Microbiological Outcomes

The clinical success rate of TGC therapy was 48.4% (*n* = 15). TCG therapy failed in 16 (51.6%) patients: ten patients died during TGC therapy, and six had to be switched to other antibiotics (piperacillin-tazobactam with amikacin and fosfomycin in four patients and meropenem with amikacin and fosfomycin in two patients) because their infection progressed after they had been treated with TGC for 5–11 days. The overall ICU mortality rate was 45.2% (*n* = 14). One patient died because of a complicated pulmonary embolism, and the remaining thirteen died because of deteriorated pneumonia.

Microbiological eradication was achieved in 19 patients (61.3%). Persistent positive culture results were found in the other 12 patients (38.7%) at the end of TGC treatment (Table 1).

APACHE II scores were lower (15.0* versus* 19.0, *P* = 0.04), the duration of IIAT was shorter (7 days* versus* 16 days, *P* = 0.02), and the rate of microbiological eradication was higher (13 cases* versus* 6, *P* = 0.009) in successfully treated patients than in unsuccessfully treated patient. No differences were observed in demographic features, comorbidities, SOFA scores, antibiotics used prior to TGC, or the duration of TGC therapy (Table 1).

The clinical success rate was higher [65.0% (13/20) versus 18.2% (2/11), *P* = 0.023] and the ICU mortality rate was lower [30.0% (6/20) versus 72.7% (8/11), *P* = 0.031] in the patients who received a high-dose TGC therapy (100 mg every 12 h after a 200 mg loading dose) than in patients who received a standard-dose TGC therapy (50 mg every 12 h after a 100 mg loading dose). No significant difference was observed in the microbiological eradication rate between the two different doses of TGC therapy [45.0% (9/20) versus 27.3% (3/11), *P* = 0.452].

## 4. Discussion

In this study, we found that the clinical cure rate for patients treated with TGC regimens was 48.4% in a cohort of critically ill patients with nosocomial pneumonia who had received IIAT as a result of CRGNBs. The rate of microbiological eradication was 61.3% and the overall ICU mortality rate was 45.2%. APACHE II scores were higher and IIAT durations were longer in unsuccessfully treated patients than in successfully treated patients. The high-dose TGC therapy (100 mg every 12 h after a 200 mg loading dose) was superior to the standard-dose TGC therapy (50 mg every 12 h after a 100 mg loading dose).

IIAT has been shown to significantly increase the likelihood of morbidity and mortality in patients with nosocomial pneumonia, and antibiotic resistance is the main cause of IIAT [14, 19, 20]. In Asia, the major isolates obtained from cases with HAP and VAP are nonfermenters (e.g.,* Acinetobacter* and* Pseudomonas aeruginosa*) and Enterobacteriaceae [19, 20]. The rate of imipenem resistance in* Acinetobacter* is 67.3% in Asian countries, while the rates for MDR and extensive drug resistance are 82% and 51.1%, respectively. The MDR rate for* Klebsiella pneumoniae* is 44.7% [20]. Consistent with these data, in our study, 67.7% of the carbapenem-resistant isolates were* A. baumannii*, and the others were* K. pneumoniae* and* E. coli*.

Our study is novel because it was focused on patients with HAP/VAP who were treated with a TGC regimen as a salvage therapy subsequent to the failure of a previously selected antibiotic because of carbapenem-resistant pathogens. The off-label use of TGC has become widespread because of the scarcity of approved effective alternative antibiotics for MDR infections, such as nosocomial pneumonia, in addition to bloodstream and urinary tract infections [21]. In a few retrospective studies, TGC has demonstrated good efficacy against HAP and VAP caused by CRGNB. These studies have shown that TGC was effective in most patients (69.7–90%) with VAP caused by carbapenem-resistant* A. baumannii* when used alone or in combination with other antimicrobials [12, 13, 22]. For* E. coli* and* Klebsiella* spp., which exhibit decreased susceptibility to carbapenems, the in vitro rates of susceptibility to TGC are 100% and 94.8%, respectively. Nearly 70% of patients who are treated with TGC achieve resolution when an infection was caused by a carbapenem-resistant or MDR Enterobacteriaceae [2].

A recent multicenter clinical study of VAP patients reported a significantly lower cure rate for a TGC regimen than was observed for an imipenem regimen [23]. However, only approximately 30% of the patients experienced a prior antibiotic failure in this previous study, and this rate is significantly different from the rate observed in our cohort. A meta-analysis including 13 randomized clinical trials demonstrated that TGC was associated with an increase in mortality and an increase in the noncure rate. These effects were independent of the infection type, trial design, and study size [7]. However, TGC has been prescribed in life-threatening infections for which there were few or no alternative agents [9–11, 15, 24]. A high-dose TGC regimen has been shown to result in better outcomes in patients with severe infections caused by MDR bacteria [15]. Our data also indicate that a high-dose TGC therapy is superior to a standard-dose TGC therapy. Pharmacokinetic-pharmacodynamic properties and patient-specific factors appear to offer one explanation for the observed differences in outcomes in TGC-treated patients with HAP. An inferior pharmacokinetic-pharmacodynamic index was observed in patients with poor clinical and microbiological responses [25]. Lower albumin concentrations, VAP status, worsening infections, or complications with baseline bacteremia have been associated with poor clinical outcomes [25, 26]. Additionally, in our cohort, higher APACHE II scores and a longer duration of IIAT were observed in unsuccessfully treated patients.

Our study has several limitations. First, we performed a retrospective analysis that included a small number of patients. Second, in almost all of the patients, TGC was used in combination with other antibiotics. The small number of cases included in this study did not allow the use of a regression model that would separate the effect of TGC alone from the combined effects of the other antibiotics or the effects of the clinical characteristics, such as the APACHE II score. Finally, we did not monitor plasma or tissue concentrations of TGC. However, to the best of our knowledge, this is the first study to focus on TGC as a salvage regimen for HAP caused by CRGNB.

## 5. Conclusions

TGC, especially a high-dose regimen, might be a justifiable option in critically ill patients with nosocomial pneumonia caused by CRGNB who received IIAT for whom options are limited. Multicenter, prospective, randomized controlled clinical trials and pharmacokinetic research are required to confirm the role of TGC as an effective salvage treatment in critically ill patients with serious CRGNB infections.

## Figures and Tables

**Table 1:** Demographic and clinical data for the included patients.

Variable^**∗**^	Total (*n* = 31)	Successful cases (*n* = 15)	Unsuccessful cases (*n* = 16)	*P* value^#^
Age, years, mean (SD)	74.6 (9.4)	74.8 (9.6)	74.4 (9.8)	0.93
Male, *n* (%)	22 (71.0)	13 (86.7)	9 (56.3)	0.113
APACHE II score, median (IQR)	19.0 (15.0–19.0)	15.0 (13.5–19.0)	19.0 (18.0–20.0)	0.04
SOFA score, mean (SD)	3.5 (1.1)	3.1 (0.8)	3.8 (1.2)	0.16
Severe sepsis/septic shock, *n* (%)	11 (35.5)	5 (33.3)	6 (37.5)	1.0
VAP, *n* (%)	10 (32.3)	4 (26.7)	6 (37.5)	0.704
ICU LOS before TGC, day, median (IQR)	8 (1–13)	7 (0.5–10.0)	9 (6.3–14.5)	0.16

Comorbidities, *n* (%)				
COPD	16 (51.6)	7 (46.7)	9 (56.3)	0.724
Bronchiectasis	3 (9.8)	1 (6.7)	2 (12.5)	1.0
CHF	8 (25.8)	5 (33.3)	3 (18.8)	0.433
Diabetes	5 (16.1)	1 (6.7)	4 (25.0)	0.333
Cerebral apoplexy	3 (9.8)	2 (13.3)	1 (6.25)	0.60
Immunosuppressive status	5 (16.1)	3 (20.0)	2 (12.5)	0.654
Comorbidities >1	17 (54.8)	10 (66.7)	7 (43.8)	0.285

Responsible pathogens, *n* (%)				
*Acinetobacter baumannii*	21 (67.7)	9 (60.0)	12 (75.0)	0.458
*Klebsiella pneumoniae*	5 (16.1)	3 (20.0)	2 (12.5)	0.654
*Escherichia coli*	3 (9.7)	1 (6.7)	2 (12.5)	1.0
*Stenotrophomonas maltophilia*	2 (6.5)	0	2 (12.5)	0.484

Type of initial empiric antibiotics, *n* (%)				
Cephalosporin	4 (12.9)	1 (6.7)	3 (18.8)	0.60
Combination *β*-lactam/*β*-lactamase inhibitor	12 (38.7)	6 (40.0)	6 (37.5)	1.0
Carbapenem	15 (48.4)	10 (66.7)	5 (31.3)	0.076
Fluoroquinolone	9 (29.0)	4 (26.7)	5 (31.3)	1.0
Aminoglycoside	8 (25.8)	6 (40.0)	2 (12.5)	0.113
Glycopeptide	5 (16.1)	1 (6.7)	4 (25.0)	0.333
Duration of initial treatment with antibiotics, day, median (IQR)	13 (7–20)	7 (7–14)	16 (7–20)	0.02
Duration of treatment with TGC, day, mean (SD)	14.3 (2.8)	12.7 (5.9)	15.5 (4.2)	0.51
Microbiological eradication, *n* (%)	19 (61.3)	13 (86.7)	6 (37.5)	0.009
ICU mortality, *n* (%)	14 (45.2)	1 (6.7)	13 (81.3)	0

^*∗*^APACHE II: Acute Physiology and Chronic Health Evaluation II, CHF: chronic heart failure, COPD: chronic obstructive pulmonary disease, ICU: intensive care unit, IMV: invasive mechanical ventilation, IQR: interquartile range, TGC: tigecycline, LOS: length of stay, SD: standard deviation, SOFA: Sequential Organ Failure Assessment, and VAP: ventilator-associated pneumonia.

^#^Successful cases versus unsuccessful cases.

**Table 2:** Rates of resistance (%) of the isolates to antimicrobial agents.

Antimicrobial	*Acinetobacter baumannii *(*n* = 21)		*Klebsiella pneumoniae *(*n* = 5)		*Escherichia coli* (*n* = 3)		*Stenotrophomonas maltophilia *(*n* = 2)	
	*I* ^*∗*^	*R* ^*∗*^	*I*	*R*	*I*	*R*	*I*	*R*
Ampicillin	0	100				100		
Piperacillin-tazobactam	9.5	76.2	0	80.0	33.3	66.7	0	100
Ceftriaxone	0	100	0	100	0	100		
Ceftazidime	4.8	81.0	20.0	80.0	0	100	0	100
Cefepime	19.0	76.2	20	60.0	33.3	66.7		
Cefoperazone-Sulbactam	14.3	476.2	0	100	33.3	66.7	0	100
Aztreonam	28.6	71.4	0	80.0	0	66.7	0	100
Imipenem	23.8	66.7	25.0	75.0	0	100	0	100
Meropenem	28.6	61.9	0	100	33.3	66.7	0	100
Levofloxacin	33.3	57.1	20	60.0	0	100		
Minocycline	28.6	52.4	20	60	0	66.7		
Gentamycin	14.3	47.6	0	50	0	100	50	50
SMZ-TMP	0	47.6	0	80			0	50
TGC^*∗*^	4.8	0	0	0	0	0	0	0

^*∗*^TGC: tigecycline, *I*: intermediate, and *R*: resistance.
